# Pediodontia*Read before the National Dental Association at its twenty-third annual session, New Orleans, La., October 20-24, 1919. Reprinted from the *Journal of National Dental Association*.

**Published:** 1921-01

**Authors:** P. R. Thomas

**Affiliations:** Minneapolis, Minn.


### PEDIODONTIA*

BY P. R. THOMAS, D.D.S., MINNEAPOLIS, MINN.

In presenting for the consideration of the dental profession a paper on Pediodontia (children's teeth), I am actuated by a desire to call attention to that most neglected part of our population, the little children between the ages of two and six years. From a dental standpoint, little attention has been paid them. The condition of this part of our population is so closely related to the dental conditions in our schools that it merits serious consideration. We are confronted by statistics gathered in our schools from time to time, showing the terrible conditions found among the children examined from a dental standpoint. For the correction of these conditions, education of the children, establish-

* Read before the National Dental Association at its twenty-third annual session, New Orleans, La., October 20-24, 1919. Reprinted from the *Journal of National Dental Association.*

ment of clinics, instruction in the use of the toothbrush, and other means have been advocated and used. It is from but few parts of the country, however, that the results of this work have been reported.

The reason for the lack of more widespread work along this line has been the inability to educate the proper authorities as to the value of the work, as well as a lack of funds which might be appropriated for dental clinics. We no sooner make a start in the work of repairing the teeth of children in schools, and begin to make progress, before a new influx of children, with equally bad mouth conditions, confronts us. To care for these new patients, in addition to keeping up with the repair work among the older children, previously treated, is a monumental task.

Year after year this condition does and will confront us, and something must be done to get the new pupils started in school with better mouth conditions. In all school and institutional work we are hampered by a lack of operators. However, this phase of the problem has been much bettered by the entrance of the oral hygienist into the field. It will take years before oral hygienists in sufficient numbers can be educated to carry on this important work.

In addition to all the means being used to relieve the situation confronting the dental profession, have we not neglected a most important factor, which might change the status of affairs and greatly help in this struggle against diseased conditions in the child's mouth? Can we not approach the problem from the standpoint of bringing the children up to school age with better mouth conditions than is at present the case? In my judgment, the greatest aid in the betterment of mouth conditions in our schools will be found in the education and care the mother can give the child between the ages of two and six years, so that when the child enters school, it will do so with greater freedom from caries, and the consequent destruction of tooth structure. Granting, then, that the mother, caring for the health of the child, is the logical caretaker of the child's teeth, what can we do to educate the mother?

The expense of buying toothbrushes, without doubt, deters thousands of mothers with large families from properly caring for the teeth of their children. For the reason, then, that we must aid the children of the poor, as well as the children of the rich, a cheap means of caring for the children's teeth must be demonstrated to the mother. As a means to this end, and on account of the cheapness of the materials required, the use of orange or bass-wood sticks with a dentifrice, or some cheaper material, such as precipitated French chalk, can be recommended; and for polishing the interproximated surfaces of the teeth, fine white wool yarn, or any other carrying medium, will suffice.

We have at hand, then, materials for caring for children's teeth, easily within the means of all mothers, for the care of all the children. The education of the mother by the dentist, or oral hygienist, should cover many things of importance in relation to the care of the teeth, and the building up of a normally healthy mouth. Having devoted considerable time to this kind of educational work, I believe the mother should be taught the following, among many other things, with regard to a child's mouth:

First, the value of the deciduous teeth as related to the child's health and development. The health and development of the child depends upon its ability to play well, sleep well and eat well. That the lack of desire to play, to sleep and to eat is induced more by aching, diseased teeth than all other factors combined. That the structure and occlusion of the permanent teeth is largely dependent on the preservation intact of the deciduous teeth.

Second, that the care and preservation of the child's teeth is dependent upon the mother's efforts, for the child will permit the mother to care for it where it will not permit a stranger to do so. That the preservation of a healthy, wholesome mouth condition is the result of systematic, thorough and regular care, carried out in detail.

Third, that the surfaces most vulnerable to decay in the child's mouth are the occlusal surfaces of the molars and the interproximate surfaces of the teeth.

Fourth, that as a supplement to the proper daily use of the toothbrush, all the surfaces of the teeth should be polished at least once a month with an orange or basswood stick, pumice or powder, and the interproximate surfaces polished with flat floss silk, or fine white wool yarn. That this will protect the child's teeth between 85 and 95 per cent. against caries, if thoroughly and regularly done.

Fifth, that as an aid in the prevention of caries, the occlusal surfaces of the deciduous molars should be protected with cement in the same manner that Dr. G. V. Black advised the protection of the six-year molar.

Sixth, that the six-year molar is not a temporary tooth. That it is not replaced by another tooth, if lost, and that erupting as it does, in early childhood, it must be especially the object of care and should be protected by the use of cement rubbed onto the occlusal surface as soon as the tooth erupts.

Seventh, that the normal development of the jaws, preparatory to the eruption of the permanent teeth, is dependent largely on the development of the muscles of mastication. For this reason the child should thoroughly masticate all its food. That the food should be prepared in such a way as to require thorough mastication.

Eighth, that according to Dr. Dewey, in his work entitled "Practical Orthodontia," five of the nine acquired causes of malocclusion are due to the loss of deciduous tooth structure. That the results of habits of childhood, such as thumb-sucking, tongue-sucking, and the use of pacifiers, are destructive to normal occlusion.

Ninth, that candy, and carbohydrates in other forms, is the worst enemy of sound teeth, for the reason that the destruction of the teeth by caries is most easily induced by chemical changes in these substances, lodged about the teeth.

Tenth, that a thorough knowledge of a good technic in the use of the toothbrush is one of the greatest means known for the preservation of the teeth. That the child should not be depended upon to protect its own teeth, through its own efforts, but that the use of the brush, in the hands of the mother, in the care of the child's mouth, is absolutely essential in early childhood.

The technic of hand-polishing the surfaces of the teeth should be carefully taught, for it is only by systematic care that the desired results can be obtained. At regular times, and regular intervals, thorough brushing of the teeth, and prophylaxis carried out in detail is essential in order to prevent the beginning of tooth destruction, for prevention must be the ultimate goal.

Observation makes it clear that the dental profession of today is not fulfilling its duty in the dental education of the public, and particularly of the mothers. There is no widespread, systematic effort being made to carry much-needed information to all parts of the country. What we need is a strong, well-financed organization to carry on this much-needed educational propaganda. In oral hygiene work lies the dentist's greatest opportunity for good, and it is up to the dental profession. Thoroughly systematized, this work can be of inestimable value to all people, especially in its relation to the health and development of the children.

No time can be used to better advantage than in helping educate the mothers of America, by teaching them methods useful in preserving the children's teeth. In closing, let me emphasize the necessity for the fullest co-operation of every member of the profession in this work. Dentistry for children must play a leading part in the health work among the children of our country. Remembering that a normal, healthy mouth is the true basis of good health, may we not all be inspired to greater effort in this important cause. A normal mouth with a full complement of prophylactically clean teeth is splendid health insurance.—*Journal N. D. A.*, October, 1920.

